# New Non-Linear Color Look-Up Table for Visualization of Brain Fractional Anisotropy Based on Normative Measurements – Principals and First Clinical Use

**DOI:** 10.1371/journal.pone.0071431

**Published:** 2013-08-22

**Authors:** Jiří Keller, Aaron M. Rulseh, Arnošt Komárek, Iva Latnerová, Robert Rusina, Hana Brožová, Josef Vymazal

**Affiliations:** 1 Department of Neurology, Third Faculty of Medicine, Charles University in Prague, Prague, Czech Republic; 2 Department of Radiology, Na Homolce Hospital, Prague, Czech Republic; 3 Department of Probability and Mathematical Statistics, Faculty of Mathematics and Physics, Charles University in Prague, Prague, Czech Republic; 4 Department of Neurology, Thomayer Hospital, Prague, Czech Republic; 5 Department of Neurology and Center of Clinical Neuroscience, First Faculty of Medicine and General University Hospital in Prague, Prague, Czech Republic; University Of Cambridge, United Kingdom

## Abstract

Fractional anisotropy (FA) is the most commonly used quantitative measure of diffusion in the brain. Changes in FA have been reported in many neurological disorders, but the implementation of diffusion tensor imaging (DTI) in daily clinical practice remains challenging. We propose a novel color look-up table (LUT) based on normative data as a tool for screening FA changes. FA was calculated for 76 healthy volunteers using 12 motion-probing gradient directions (MPG), a subset of 59 subjects was additionally scanned using 30 MPG. Population means and 95% prediction intervals for FA in the corpus callosum, frontal gray matter, thalamus and basal ganglia were used to create the LUT. Unique colors were assigned to inflection points with continuous ramps between them. Clinical use was demonstrated on 17 multiple system atrophy (MSA) patients compared to 13 patients with Parkinson disease (PD) and 17 healthy subjects. Four blinded radiologists classified subjects as MSA/non-MSA. Using only the LUT, high sensitivity (80%) and specificity (84%) were achieved in differentiating MSA subjects from PD subjects and controls. The LUTs generated from 12 and 30 MPG were comparable and accentuate FA abnormalities.

## Introduction

Radiologists primarily make clinical decisions based on the qualitative assessment of images. A general impression may be followed by measurements of size (e.g., of a solid tumor), quantitative tissue measures (e.g., apparent diffusion coefficient in acute stroke) or by more complex post-processing (e.g., in cardiac magnetic resonance imaging). Despite the fact that the Hounsfield unit (based on the amount of X-radiation absorbed by tissue, used in computed tomography) is no longer the only absolute voxel intensity measure and widespread film-less radiology enables comparison of quantitative parameters with previous records, voxel value measurements in magnetic resonance imaging (MRI) remain uncommon.

Diffusion tensor imaging (DTI) has become a useful quantitative tool in experimental medicine for investigating certain morphological and functional characteristics of the brain in both health and disease states [1]–[3]. Scalar invariants may be derived from the tensor to characterize diffusion, with fractional anisotropy (FA) and mean diffusivity (MD) being the most commonly reported absolute measures. In some diseases typically affecting the white matter of the brain, DTI may improve the diagnostic process [4] and has shown utility in monitoring disease progression [5]–[7]. Although changes in FA have been reported in many neurological disorders [8], [9], these changes are often discrete and difficult to distinguish. Despite many reports of statistically significant results, difficulties remain in incorporating these findings into clinical practice. A new approach is needed to provide an easy tool for the evaluation of FA images. As in conventional radiology, quickly scanning for major changes in the whole volume (in a qualitative manner) could then be followed by more precise region of interest (ROI) methods to assess local FA changes. We propose a new color look-up table (LUT), based on the statistical analysis of normative FA data, to assist in the quantitative assessment of FA images in the clinical setting.

Attempts to use a color LUT in traditional MRI were presented more than twenty years ago. Weiss et al. [10] based their LUT on intensities in T1- and T2-weighted images, which were used as luminance and hue values. They were followed by a three-parametric approach by co-registering T1, T2 and T2 Fluid Attenuated Inversion Recovery (FLAIR) images. Intensities in each of the images were used as color-component values of corresponding voxels (matching signal intensities in respective weightings with red, green and blue (RGB) components) [11]. Colors in these scales do not represent exact T1 or T2 relaxation times and therefore do not allow quantification of MRI measures. Generating such images requires co-registration of the acquired images using different sequences, taking into account potential patient movement, different contrast and potentially different resolution as well. To achieve this, balancing the parameters of the sequences to be as similar as possible would be beneficial, but would result in an unacceptable decline in quality in some of the sequences. Moreover, difficulties arise in the windowing of color images in general, as the control of the center of the window and window width are more intuitive with a black and white LUT. Due to the mentioned limitations, color maps have not become a routine part of clinical practice, with the exception of perfusion and directionally-encoded color diffusion maps [12]. These maps facilitate the evaluation of principal fiber orientation and the detection of abnormal orientation of the principal eigenvector.

Aside from the directionally-encoded maps (which are modulated by FA, but not intended to be used for FA evaluation purposes), FA is sometimes color-encoded by a linear scale. This is done mainly for presentation purposes and the resulting images rarely emphasize the anatomy or pathology. To the best of our knowledge, a nonlinear LUT is not currently used in MRI.

To test the clinical utility of the new scale, we included a group of patients with multiple system atrophy (MSA). MSA is a neurodegenerative disorder of the CNS characterized by globally reduced FA [13], with maximal changes involving the medial cerebellar peduncle (MCP) [14], pons and internal capsule (IC) [15]. MSA shares some common clinical features with Parkinson disease (PD), but PD does not exhibit decreased FA in the MCP, pons or IC. Color change in these regions may therefore be potentially useful for differentiating MSA patients from PD patients, as well as from other neurodegenerative disorders with normal FA in above-mentioned areas (e.g., progressive supranuclear palsy).

We designed a versatile LUT for the fast and easy detection of FA changes. The proposed LUT was tested by means of sensitivity and specificity on a disease with known FA decline. The proposed novel algorithm can also be used to build a site-specific LUT, reflecting different signal-to-noise ratios (SNR) in different hardware and software environments (e.g., different manufacturer, magnetic field strength).

## Methods

### Participants

Normative data are based on FA values measured in 76 volunteer subjects determined to be free of neurological disease, aged 15–80 years (41 male, 35 female; mean age 44.4±18 SD). In a subgroup of 59 subjects (31 male, 28 female; mean age 43.8±18 SD), a sequence with 30 motion-probing gradient (MPG) directions was additionally acquired in the same scanning session.

To validate potential clinical applicability, we applied the LUT to an anonymized set of 17 MSA patients, 17 age- and sex-matched healthy volunteers and 13 patients with PD. For this validation we used the 12 MPG sequence.

The entire study was approved by the Ethics committee of Na Homolce Hospital and all participants or their guardians provided signed, informed consent (applies to all cohorts in the present study). Source datasets cannot be made publicly available to maintain the privacy of the subjects.

### Data acquisition and preprocessing

All imaging was performed on a Siemens Avanto 1.5 Tesla System equipped with Quantum gradients (Erlangen, Germany) and fitted with a 12-channel 4-segment head coil. T1WI, T2WI, FLAIR, and T2^*^-weighted structural magnetic resonance images were acquired to exclude pathology. Diffusion weighted imaging was performed in 12 and 30 non-collinear motion-probing gradient (MPG) directions (b = 1100; TR = 8839/8800 ms, TE = 95/98 ms, NEX = 2; Matrix = 128×128; FOV = 280 mm; Bandwidth = 1345 Hz/px; GRAPPA = 2; voxel size 2.2 mm isotropic). FA data can be obtained either directly from the magnetic resonance scanner in the DICOM format or calculated offline in stand-alone software. We processed diffusion weighted images offline in FSL (FMRIB Software Library [16]). First, NIFTI-converted data were subjected to eddy-current and motion correction, followed by brain extraction using BET (brain extraction tool) [17]. Dtifit (part of FSL) was then used to calculate the scalar invariants of the tensor. Dtifit generates 3D images at the same matrix size and resolution as the original diffusion images, including a raw T2-signal image with the same distortions as the diffusion weighted images.

### FA measurement

**Figure 1:**
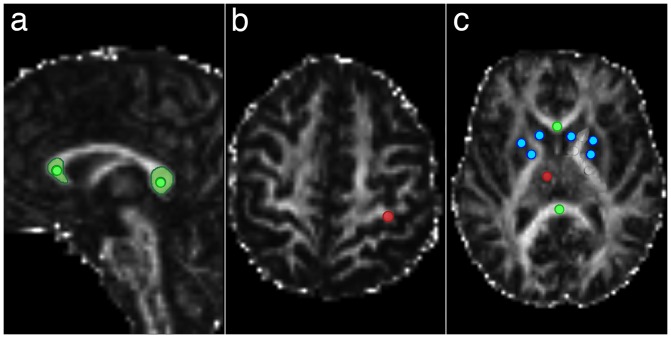
Three groups of regions of interest (ROI) used in the generation of the non-linear color look-up table (LUT). The corpus callosum (labelled green) was selected using both freehand (two ROIs) and circular ROIs (cROIs, four areas). Freehand selection included the rostrum and part of the genu of the corpus callosum as well as the splenium of the corpus callosum and were drawn in the sagittal plane (a). Circular ROIs were positioned in the middle of these anatomical areas, and were placed in the sagittal (a) and transverse (c) planes. Gray matter (red) selections included the precentral gyrus (Brodmann area 4, section (b)) and the thalamus (c). Selections in the basal ganglia (blue) were positioned in putamen, pallidum and caudate nucleus (c). The anterior aspect of the subject is on the left (a) or the top (b,c) of the images.

FA was measured using ImageJ viewer [18], with freehand regions of interest (ROI) or circular regions of interest (cROI) placed by a single observer (A.R). Circular regions of interest measured 15 mm^2^ and were positioned in the center of the structure to measure the most compact part. Several white and gray matter regions of the brain that exhibit various FA values and can be affected in some disease states were included: the corpus callosum (CC), gray matter (GM, precentral gyrus and thalamus) and basal ganglia (BG). Freehand ROIs used for the generation of the scale were placed in the rostrum and genu of the corpus callosum (for anatomical reference see Figure 1 in [19]). The placement of ROIs is shown in Figure 1 (for a list of ROIs, see figure legend). To achieve standard positioning of the ROIs, we used the midsagittal and transverse planes at the level of the posterior thalamus and at the level where the subcortical white matter of the precentral gyrus was best visible. In the areas of lower anatomical resolution (basal ganglia and precentral gyrus), ROIs were first placed on the T2-weighted image generated by DTIFit and then projected on the corresponding FA map. Other selections were placed directly on the FA maps. A total of 16 ROIs were selected and used for the generation of the LUT, 14 of which were cROIs (in total, 1216 FA values). For validation purposes, the same methodology was used for the measurement of FA in the pyramidal tract and in the middle cerebellar peduncles.

### Preparation of the LUT

For each ROI group (e.g., CC), several ROIs (e.g., anterior and posterior part) were measured in each subject. These measurements were considered repeat may enable the detectionobservations of the particular ROI group (e.g., CC), as they are not independent. To account for these dependencies, a random intercept linear mixed model [20] was fitted in each ROI group. Based on the fitted model, we estimated the ROI group-specific (e.g., for the whole CC) expected value (population mean) and the 95% prediction interval (PI). Statistical analysis was performed using R software, version 2.12.2 [21] and the R package lme4 [22].

**Figure 2:**
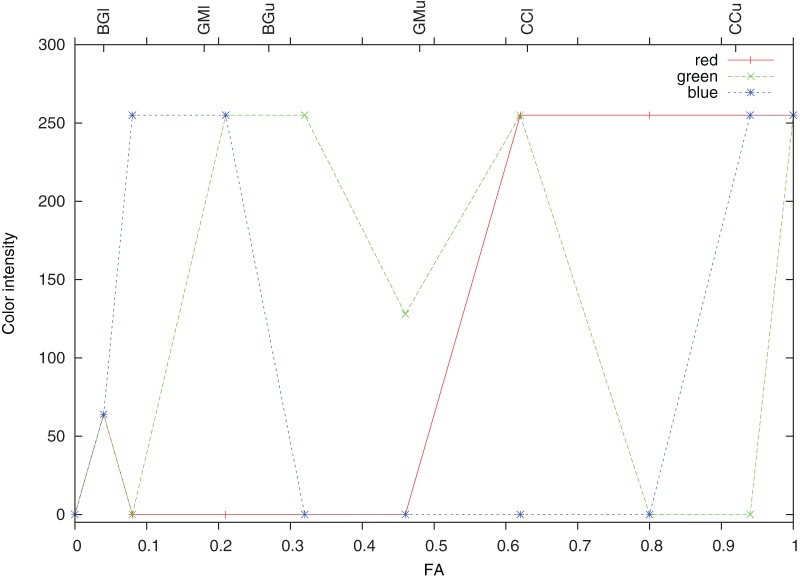
Definition of the color look-up scale for fractional anisotropy (FA). The color intensity for red, green and blue channels is in the range of 0–255, the combination of the intensities in these channels results in a unique color for any FA value.

**Figure 3:**
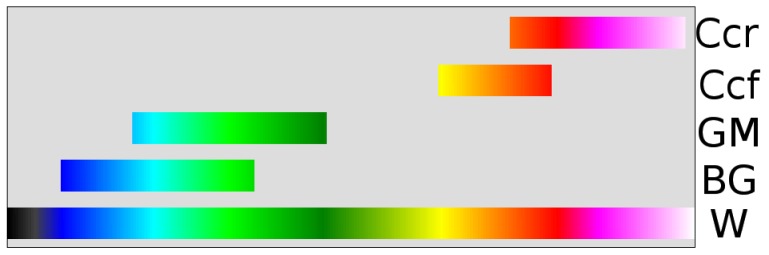
Fractional anisotropy in regional groups. W, whole scale; BG, basal ganglia; GM, gray matter; Ccf, freehand selection in the corpus callosum; Ccr, circular region of interest in the corpus callosum.

Results of descriptive statistics were used as inflection points, which were assigned unique colors, and continuous ramps were generated (see Figure 2) to create a color transition between them (see Figure 3). Based on these ramps, FA values were assigned unique colors.

After creating a LUT for 12 MPG data, the same process was applied to a subgroup of 59 subjects that were additionally scanned in 30 MPG.

### Method of validation

For validation of the potential clinical use, an anonymized set of MSA patients, healthy volunteers and PD patients was presented to four blinded readers (J.V., 20 years of practice, J.K., 5 years of practice, A.R, 2 years of practice, I.L., novice to reading MRI). Readers were asked to assess datasets as MSA or non-MSA mainly using FA information from the MCP, pons, pyramidal tract and basal ganglia, as FA changes have been reported in these regions (for references see Introduction). No other images were presented.

## Results and Discussion

### Generation of the LUT

**Figure 4:**
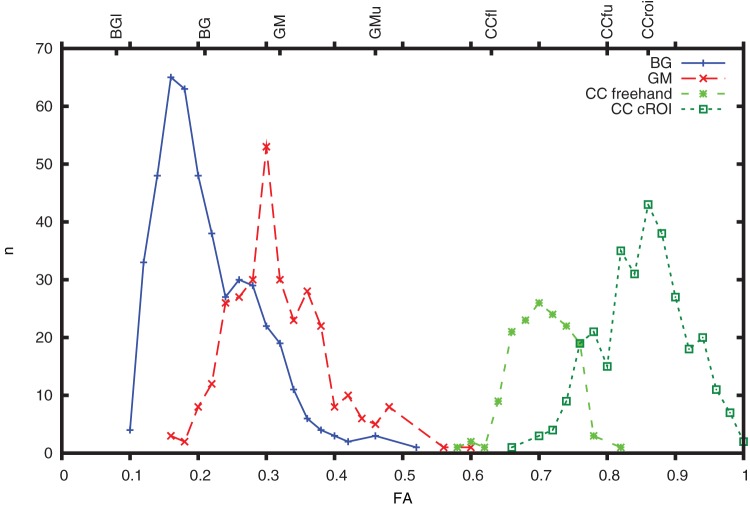
Histogram of fractional anisotropy values in the groups of regions of interest (ROI) used to generate the color scale. BG, basal ganglia; GM, gray matter; CC freehand, freehand selection in the corpus callosum; CC cROI, circular ROI selection in the corpus callosum.

**Figure 5:**
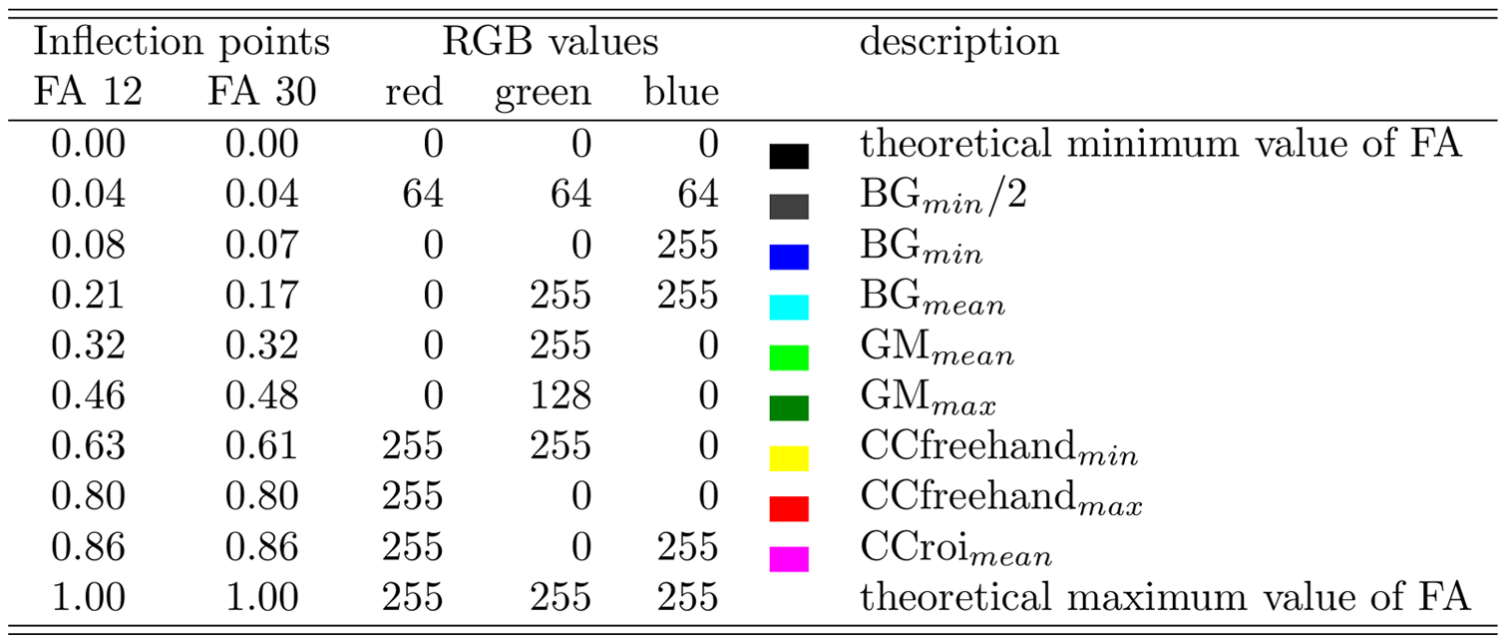
Inflection points for the Look-Up Table. FA, fractional anisotropy; RGB, red-green-blue components of color (note: this image is in CMYK colorspace, therefore colors do not exactly match RGB colorspace); BG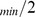, lower border of prediction interval for basal ganglia (BG) divided by two; BG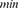, lower border of prediction interval for BG; BG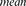, mean value for BG; GM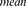, mean value for gray matter (GM); GM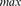, upper border of prediction interval for GM; CCfreehand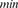, lower border of prediction interval for freehand selection of corpus callosum (CC); CCfreehand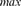, upper border of prediction interval for freehand selection of CC; CCroi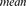, mean value for circular selection of CC.

A nonlinear LUT was created using inflection points based on FA values in the BG, GM and CC. The results of descriptive statistics for these regions, including both 12 and 30 MPG, are shown in Table 1. The mean FA of white matter ROIs was equal in both sequences, while FA values in the BG and GM differed. A group histogram of FA in the regions used in generating the scale (Figure 4) shows normality. Inflection points are presented at the top of the histogram and their FA values are listed in Figure 5. Color ramps (see Figure 2) were used to define the LUT (see Figure 3).

**Table 1:** Estimated mean values and prediction intervals (PI).

Group	12 MPG		30 MPG	
	Mean	PI	Mean	PI
Basal ganglia	0.218	(0.077, 0.359)	0.173	(0.073, 0.273)
Gray matter	0.323	(0.181, 0.464)	0.328	(0.175, 0.481)
CC freehand	0.709	(0.627, 0.792)	0.703	(0.608, 0.797)
CC_cROI_ total	0.858	(0.731, 0.986)	0.855	(0.717, 0.992)
genu CC	0.817	(0.712, 0.923)	0.812	(0.685, 0.939)
splenium CC	0.899	(0.805, 0.994)	0.897	(0.806, 0.989)

CC, corpus callosum; cROI, circular region of interest.

The corpus callosum was included as it is one of the most robust white matter tracts with compact fiber bundles (providing very high FA values), and FA changes in the CC have been reported in many disease states (e.g., Alzheimer disease [23], multiple sclerosis [24], migraine [25] and neglect syndrome [26]). We included both freehand and cROI measurements as freehand selections cover a broader range of values. Freehand selections had lower average values than those obtained by cROI, which represent the core of packed fiber bundles (see Figure 3). FA in the GM was substantially lower than in the white matter, and overlapped with values recorded in the basal ganglia. Although FA is not as commonly measured in GM regions as in the white matter (tensor fitting becomes less reliable in low FA regions), our LUT may enable the detection of FA changes in the GM, which may be of use in some neurodegenerative diseases (e.g., amyotrophic lateral sclerosis [27] or Huntington disease [28]). However, one should keep in mind that higher inter-individual variability in diffusion indices in these regions is not independent of signal to noise ratio (SNR), especially in the basal ganglia, where tissue iron substantially influences FA values [29].

To the best of our knowledge, we present the first non-linear LUT based on the statistical assessment of data acquired using an ROI-based approach. We elected to use this approach, versus a voxel-based approach, as it does not include smoothing of the data which may decrease FA by partial voluming from neighboring voxels and may therefore better reflect real FA in the selected structure. It has been reported that voxel-based and ROI-based methods give complementary, rather than identical, results [30], [31]. The measurements obtained with cROIs are valid only for included voxels and cannot be directly generalized for the whole anatomical structure. To account for this, we used several cROIs to characterize the respective anatomical structures, and used both the mean and prediction intervals to create the LUT. Higher values in cROI than in freehand selection reflect the definition of the area of interest (the highest FA region in the anatomical structure). The freehand selections included not only the most dense core of the tract but also the heterogeneous periphery, containing radiating fibers which have lower FA.

The color scale is based on FA values in three anatomical areas. The inflection points for the LUT are not intended as absolute values for a particular structure but rather as a value for particular white matter density. Therefore, to reflect areas with higher fiber density, we merged data from the splenium and genu of the corpus callosum, even though they differ.

Although FA is dependent on age in many parts of human brain [32], our data cover nearly the entire extent of adulthood and age correction was applied. For potential use in the pediatric population, brain development has to be taken in account and several age-specific scales may be needed.

### Validation

#### Other regions of interest

**Figure 6:**
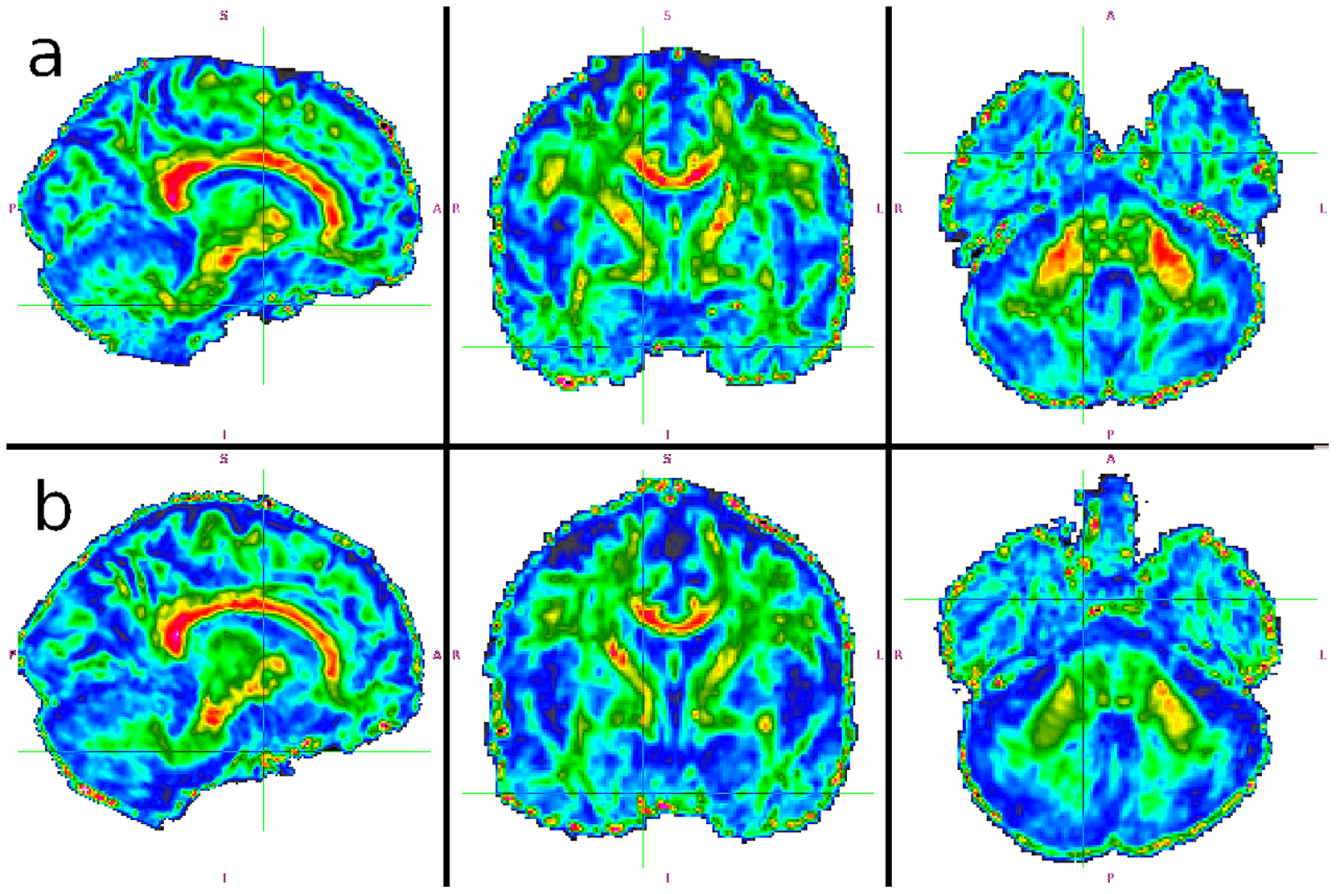
Possible application of the look-up table to fractional anisotropy imaging. a) Healthy volunteer data (60 year old male), b) A subject with multiple system atrophy (62 year old male). Decreased FA in the cerebellar peduncles is best appreciated in the rightmost image).

Using the same methodology, we additionally measured FA in the pyramidal tract and middle cerebellar peduncle. The pyramidal tract is a major white matter bundle connecting the primary motor cortex in the frontal lobe and peripheral neurons in the spinal cord. After the fibers coalesce from the frontal cortex, this structure becomes visible on the coronal view as a symmetric, yellow/red structure passing from the top lateral part of the image to the center (see the middle column of Figure 6). The pyramidal tract was selected using both cROI (FA = 0.76, PI 0.66–0.85) and freehand (FA = 0.73, PI 0.69–0.78) selections. In total (using all values), FA was 0.75 with PI 0.67–0.82. FA in freehand selection was lower than in cROI, but the overlap is significant.

The same approach was used for the middle cerebellar peduncle (best seen on the right column of Figure 6, in healthy subjects depicted in red/yellow), which is one of the three paired white matter connections of the cerebellum. These connections are crucial for movement coordination and their impairment can cause severe clinical manifestations. We used cROI (FA = 0.82, PI 0.75–0.90) and freehand selection (FA = 0.65, PI 0.56–0.73), and combining both resulted in FA = 0.73 with PI 0.54–0.93.

Both the pyramidal tract and cerebellar peduncles are major white matter bundles. In comparison with the CC, cROI values for both structures were lower (0.76 and 0.82 compared with 0.86). This is in agreement with the fact that the CC contains the most packed bundles in the brain, providing communication between the hemispheres. For the use of the LUT, it is important to note that the lower limit of the prediction interval is very close in cROI cerebellar peduncle measurements (0.75) and in CC cROI (0.73), which is used to build the scale. Therefore, both structures share a common color impression for the center of the structure. Freehand selection of the cerebellar peduncle provided lower FA values, which corresponds with the fact that it is difficult to precisely define the borders of this structure. Conversely, freehand selection of the pyramidal tract gave similar results (0.73) to CC freehand (0.71). This similarity arose due to similar fiber density, and confirms that CC inflection points as values of “typical” FA in structures containing densely packed fibers are generally valid, not only for the CC.

#### Diagnostic Use

As seen in Figure 6, FA in the middle cerebellar peduncles (best seen on the rightmost images) was decreased in the MSA subject (in the second row), i.e, it did not contain an adequate amount of red on the right side of the image, and furthermore the amount of yellow was diminished on the left. This suggests relevant FA decline in these regions. In other anatomical structures such as corpus callosum or pyramidal tract, no difference was visible.

Sensitivity and specificity for correctly classifying MSA/non-MSA subjects were measured. Their values ranged between 65/70 and 100 percent (see Table 2) and were on average 80% (sensitivity) and 84% (specificity).

**Table 2:** Sensitivity and Specificity of Respective Raters in Differentiating MSA/non-MSA.

Rater (years of practice)	Sensitivity [%]	Specificity [%]
IL (novice)	77	70
AR (2)	100	80
JK (5)	76	86
JV (20)	65	100

#### Different Signal-to-Noise Ratio

We compared the results for 12 and 30 MPG data. FA values obtained in the second experiment were equal to those from 12 MPG or differed by 1% (see columns labeled “30 MPG” in Table 1), with the exception of the BG (mean FA 0.22 in 12 MPG, mean FA 0.17 in 30 MPG). This observation can be explained by the higher vulnerability of lower FA regions to noise. For more detail information on the differences between these sequences, see the supplementary material. The stability of the scale (nearly the same results were obtained by processing two datasets with different SNR and different number of MPG) allows the use of our inflection points values in scanners employing similar sequences, as well as in datasets with slightly different SNRs (e.g., at higher field strengths). Moreover, the 12 MPG diffusion-weighted sequence we use is of reasonably short duration and offers the possibility of direct SNR measurement (both datasets are stored and can be processed separately). Sites using substantially different acquisition schemes may use our approach to build a site-specific LUT that reflects the SNR.

## Conclusion

We present a novel method of generating a LUT based on normative data. The LUT was applied to two datasets with different sequence parameters, leading to similar results even though SNR differed. However, in a case of substantially different SNR, our approach can be used to build a site-specific LUT. Application to MSA is intended as proof-of-concept and guidance for future use, nevertheless, using colored FA maps alone, four independent blinded radiologists achieved reasonable sensitivity and specificity. We assume that our LUT, combined with other imaging approaches, may improve diagnostic process not only in MSA patients. It could become a useful tool in screening for FA changes, and could significantly enhance the clinical applicability of contemporary research focused on FA changes in the diseased brain. The main advantage of the LUT presented is that it is easy to use in clinical practice (contrary to ROI measurements and other more advanced analysis methods), allowing the detection of abnormal FA on plain inspection by a trained radiologist.

## Supporting Information

Figure S1 **Bland Altman plots comparing fractional anisotropy (FA) acquired in sequences with 12 and 30 motion probing gradient directions.** FA values are multiplied by 100 BG, basal ganglia; GM, gray matter; CC, corpus callosum; cROI, circular region of interest.
(TIFF)

Table S1 **Prediction intervals and correlation coefficients between fractional anisotropy (FA) in 12 and 30 motion probing gradient directions (MPG).** Group, group of the region of interest (ROI); Pred.Low, Pred.Upp – lower border and upper border, respectively of the 95% prediction interval for the difference between FA in 12 and 30 MPGs; Cor, Cor.PValue – correlation coefficient between FA in 12 and 30 MPGs and p-value of its significance, respectively. BG, basal ganglia; GM, gray matter; Ccf. freehand selection in corpus callosum; ccROI, circular ROI in corpus callosum; cc, corpus callosum.
(RTF)

Text S1 **Comments on Bland Altman plots comparing fractional anisotropy acquired in sequences with 12 and 30 motion probing gradient directions.**
(DOC)
