# Supplementary material for: New Non-Linear Color Look-Up Table for Visualization of Brain Fractional Anisotropy Based on Normative Measurements – Principals and First Clinical Use
Source: PLoS One. 2013 Aug 22;8(8):e71431. doi: 10.1371/journal.pone.0071431 (PMC3750032; doi:10.1371/journal.pone.0071431)
Supplement: Table S1 — Prediction intervals and correlation coefficients between fractional anisotropy (FA) in 12 and 30 motion probing gradient directions (MPG). Group, group of the region of interest (ROI); Pred.Low, Pred.Upp – lower border and upper border, respectively of the 95% prediction interval for the difference between FA in 12 and 30 MPGs; Cor, Cor.PValue – correlation coefficient between FA in 12 and 30 MPGs and p-value of its significance, respectively. BG, basal ganglia; GM, gray matter; Ccf. freehand selection in corpus callosum; ccROI, circular ROI in corpus callosum; cc, corpus callosum. (RTF) [file pone.0071431.s002.rtf]

Group	Pred.Low	Pred.Upp	Cor	Cor.PValue 	
BG	-0.069	0.164	0.60	<0.001 	
GM	-0.134	0.114	0.63	<0.001 	
Ccf	-0.046	0.058	0.83	<0.001 	
ccROI	-0.092	0.097	0.75	<0.001 	
cc	-0.079	0.086 	0.90	<0.001 	
