# Supplementary material for: New Non-Linear Color Look-Up Table for Visualization of Brain Fractional Anisotropy Based on Normative Measurements – Principals and First Clinical Use
Source: PLoS One. 2013 Aug 22;8(8):e71431. doi: 10.1371/journal.pone.0071431 (PMC3750032; doi:10.1371/journal.pone.0071431)
Supplement: Text S1 — Comments on Bland Altman plots comparing fractional anisotropy acquired in sequences with 12 and 30 motion probing gradient directions. (DOC) [file pone.0071431.s003.doc]

**Comments on Bland Altman plots comparing fractional anisotropy acquired in sequences with 12 and 30 motion probing gradient directions.**

Bland Altman plots (see Figure S1) compare fractional anisotropy (FA) acquired in sequences with 12 and 30 motion probing gradient directions (MPG). Except of the basal ganglia ROIs, plot shows independency of FA value dispersion. In freehand ROI in corpus callosum, the prediction interval is approximately 0.05 in both directions, in circular ROI less than 0.10 and slightly over 0.10 in the gray matter (see Table S1). Smaller spread in the freehand selection compared to circular ROI is caused by higher number of voxels measured in this region, resulting in higher stability of the measurement. Higher spread in the gray matter is likely caused by small volume of subcortical white matter in comparison to ROI size, leading to higher variability in FA and partial voluming. This is partially balanced by the measurement of the thalamus, where the ROI is placed in the middle of the structure, without significant partial volume effects.

Broad prediction interval (over 0.15 in the upper border) in the basal ganglia deserves more detailed discussion. The placement was in the centers of the structures, but still, the dispersion is visible in higher values. When splitting back to the single ROIs, the source of this variation is identified as pallidum. Aside of this project, we studied FA values in the basal ganglia in detail, and we found strong dependency of FA on iron content (see [28]**)**. Pallidum has the highest concentration of the iron, when compared to the other measured structures, and therefore pallidal FA may be the most sensitive to increase of SNR when moving from 12 to 30 directions.
